# Laser Light Pointers for Use in Companion Cat Play: Association with Guardian-Reported Abnormal Repetitive Behaviors

**DOI:** 10.3390/ani11082178

**Published:** 2021-07-23

**Authors:** Lori R. Kogan, Emma K. Grigg

**Affiliations:** 1 Clinical Sciences Department, College of Veterinary Medicine and Biomedical Sciences, Colorado State University, Fort Collins, CO 80523, USA; 2 Department of Population Health and Reproduction, School of Veterinary Medicine, University of California, Davis, CA 95616, USA; ekgrigg@ucdavis.edu

**Keywords:** feline, laser light pointer, play, abnormal repetitive behaviors, stress, frustration, feline compulsive disorder, toy

## Abstract

**Simple Summary:**

Use of laser light pointers for feline play is popular with many cat guardians. It can be an enjoyable shared interaction and provide an easy way to encourage cats to exercise. Laser light play alone, however, does not allow cats to complete the hunting sequence; cats cannot ‘catch’ the prey. It has been suggested that this might trigger frustration and stress, both of which can contribute to compulsive behaviors. This study examined the potential relationship between the use of laser light pointers for play and the occurrence of excessive or abnormal repetitive behaviors (ARBs) often linked to diagnosis of feline compulsive disorders. Using an online, anonymous survey, we explored cat guardians’ use of laser light toys (e.g., reasons for and frequency of use) and reported ARBs. A total of 618 responses, primarily female participants from the United States, were analyzed. Nearly half (45.5%) of the respondents in our study reported using laser light toys to play with their cats, although frequency of use was low for many survey respondents. Although the statement “Laser pointers can make cats frustrated if they do not get to ‘catch’ something at the end of play” was familiar to over half of respondents (52.1%), only 35.6% of respondents reported following this advice. We found significant associations between the frequency of laser light play and the occurrence of all surveyed ARBs, apart from overgrooming. Overall, the more frequently LLP toys were used, the more likely guardians were to report ARBs in their cats. Provision of outdoor access and cat age were also significant predictors of reported ARBs: indoor-only cats, and young (1–2 years) cats were more likely to display ARBs. The strongest associations were seen for behaviors which may be connected to laser light play: chasing lights or shadows, staring “obsessively” at lights or reflections, and fixating on a specific toy. These results, although correlational, suggest that laser light toys may be associated with the development of compulsive behaviors in cats, warranting further research into their use and potential risks.

**Abstract:**

Use of laser light pointers for feline play is popular with many companion cat guardians. It can be an enjoyable shared interaction and provide an opportunity for feline exercise. Laser light play alone, however, does not allow cats to complete the hunting sequence and it has been suggested that this may trigger frustration and stress, common contributors to compulsive behaviors. This study examined the potential relationship between the use of laser light pointers for play and excessive or abnormal repetitive behaviors (ARBs) often linked to diagnosis of feline compulsive disorders. Using an online, anonymous, cross-sectional survey, we explored cat guardians’ use of laser toys and reported ARBs in their cats. A total of 618 responses were analyzed, primarily female participants from the United States. We found significant associations between the frequency of laser light play and the occurrence of all surveyed ARBs, apart from overgrooming. Provision of outdoor access and cat age were also significant predictors of reported ARBs: indoor-only cats, and young (1–2 years) cats were more likely to display ARBs. The strongest patterns were seen for behaviors which may be connected to laser light play: chasing lights or shadows, staring “obsessively” at lights or reflections, and fixating on a specific toy. Although correlational, these results suggest that laser light toys may be associated with the development of compulsive behaviors in cats, warranting further research into their use and potential risks.

## 1. Introduction

Use of laser light pointers (LLPs) for play and to stimulate species-typical hunting behavior is popular with many cat guardians. Interactive play between human and cat using an LLP would appear to meet two of the five pillars of a healthy feline environment [1]: allowing for an enjoyable shared interaction between human caretaker and their cat, and potentially providing an opportunity for exercise in the form of species-typical, normal hunting behaviors. Hyman [2] has suggested that a primary reason why cats enjoy laser light play involves the light’s motion. Citing research from Tremoulet and Feldman [3,4], he notes that the nature of the motion (e.g., frequent changes in speed and direction, appearing to ‘react’ to the cat’s movements) is a critical component of the perception of animacy (i.e., that the laser light ‘prey’ is alive). The appearance of goal-directed behavior on the part of the light (e.g., ‘avoiding’ the cat, ‘hiding’ behind furniture etc.) adds to this perception, similar to the “wolfpack effect” described by Gao et al. [5], in which multiple triangles ‘chasing’ a single moving circle are perceived as animate when the triangles remained oriented (pointed) towards the circle as it moves.

However, LLP play alone does not allow cats to complete the hunting sequence; essentially, the cat can never ‘catch’ the prey. It has been suggested that this may trigger frustration and stress, common contributors to feline behavioral problems, including compulsive behaviors [6,7]. Thus, in theory at least, using LLPs to play with cats could contribute to the development of compulsive behaviors. Compulsive behaviors are usually brought on by conflict, and appear abnormal because they are repetitive, exaggerated or sustained, and displayed out of context [8]. In cats, common signs of a compulsive disorder include oral behaviors such as overgrooming, self-directed aggression (such as chasing/chewing the tail), and hallucinatory behaviors such as staring at shadows or chasing light reflections [9].

Treatment for feline compulsive disorders (FCD) often includes the identification and removal of things that cause (motivational) conflict, frustration, and stress [9]. Complete resolution of FCDs is uncommon, although treatments (including medications) are often used to decrease the frequency or severity of the behavior, thereby increasing quality of life of the effected animal [10,11]. Providing environmental enrichment is another important component of treatment, designed to decrease stress and the risk for developing stress-related behavior problems [1,6,12,13]. A common recommendation for enrichment involves interactive play with toys. To maintain the enrichment value (in addition to the physical exercise benefits) of LLP play, while minimizing the risks of frustration, many experts recommend a modification to this type of play [14,15]. This modification consists of ending LLP play by having the light land on a small toy that resembles catchable prey (e.g., a stuffed toy mouse) [16], or a high-value food treat. In their recommendations for environmental enrichment for indoor cats, Herron and Buffington [17] list laser toys as an ‘appropriate toy’ given their ability to simulate the natural predatory sequence, but caution that the “general rule among behaviorists about light-beam games is that they should always be followed by the presentation of a treat or toy to reward the cat for the extensive ‘hunt’ and to prevent frustration” (p. 5).

To our knowledge, however, the use of LLPs for play, including the potential protective value of this recommendation for reducing the risk of frustration or the development of compulsive behaviors, has never been tested. Behavior problems are a significant risk factor for relinquishment and euthanasia of domestic cats [7,18], and even without these tragic end points, often negatively impact animal welfare, especially when they are a result of stress or anxiety [7]. Additionally, compulsive behaviors can interfere with normal behaviors and damage the human-animal bond [19]. As noted by Amat et al. [20], compulsive behaviors are among the top five cat owner complaints. The negative impact of compulsive behaviors makes it important to identify any type of activity that might increase their prevalence.

This study examines the potential relationship between the use of LLPs for play with companion cats and the occurrence of excessive or abnormal repetitive behaviors (ARBs) often linked to diagnosis of feline compulsive disorder (FCD). Our primary hypothesis was that there would be a relationship between the use and frequency of laser light play and reported ARBs. We were also interested in examining how common use of LLP play is in cat guardians; their attitudes towards this type of toy (i.e., reasons they chose to use or not use LLPs); whether or not they were familiar with the recommended modification for LLP play and employed this strategy (e.g., ending the game by providing a toy or treat); and whether or not reported ARBs had any impact on the human-animal bond.

## 2. Materials and Methods

An online, anonymous, cross-sectional survey was developed using Qualtrics (Qualtrics, Inc., Provo, UT, USA). The survey was designed, reviewed, and tested by the co-investigators and their colleagues. The survey was pre-tested by eight individuals for ambiguity and/or potentially missing or inappropriate response options, with revisions made based on the results of this testing. The final survey and study design were approved by the Colorado State University Institutional Review Board (IRB # 21-10566H). Survey respondents were recruited between February 2021–April 2021 via social media (Facebook, Twitter, Instagram).

Adult (18 years or older) participants who were the current guardians and primary caretakers of at least one adult cat (at least one year of age), and had owned the cat for at least 6 months, were recruited for the study. Guardian demographics were collected (e.g., country, age group, gender, education level, number of cats in the household). A series of questions from a published survey [21] measuring the relationship between cat and guardian were included. The Pet Relationship Scales (PRS) consists of 22 self-report items organized into three subscales, “Affectionate Companionship,” “Equal Family Member Status,” and “Mutual Physical Activity”. For this study, questions from the Affectionate Companionship subscale with an additional three items (“I consider my cat a member of the family,” “I have considered relinquishing my cat because of his/her behavior” (reverse coded), and “My cat provides me comfort during difficult times” were included. Possible total scores for the Affectionate Companionship subscale range from 8–48, with higher scores indicating a closer bond. Cronbach’s alpha for the PRS Affectionate Companionship subscale in our study was 0.755.

Next, respondents were asked a series of questions about their cat, including characteristics of the cat (such as age, sex/reproductive status, whether the cat is an indoor or outdoor cat, and whether the cat is declawed). They were next asked how often their cat displayed behaviors that might indicate compulsive tendencies (spins or tail chases, chases lights and shadows, fixates on a specific toy, stares obsessively at lights or reflections, overgrooms him/herself) [8], using a 7-point Likert scale anchored by 1 = never and 7 = multiple times a day. The survey was not directed at cat guardians experiencing behavior problems with their cats; recruitment materials only mentioned wanting to understand how people played with their cats. The list of behaviors came before any other questions besides demographic questions (and the order of the behaviors was randomized). Note that in the survey, we did not define “obsessive”, instead allowing respondents to interpret the term in the most common sense, i.e., excessive, resembling an obsession (see Supplementary Materials for the complete survey). For each such behavior noted, respondents were then asked to report how the behavior impacted them and/or the cat (e.g., negatively impacts the cat’s quality of life; negatively impacts the bond we have; does not affect me at all). They could select all answers that applied. They were next asked how easy it is to redirect their cat when doing each of these behaviors using a 4-point Likert scale (from 1 = very easy to 4 = very challenging). Guardians were also asked to indicate if their cat had ever been treated by a veterinarian for any compulsive behaviors, and if they had ever sought help for excessive fear/anxiety, aggression, or “inappropriate” elimination exhibited by their cat.

The next series of questions pertained to the use of an LLP, defined as any form of play that involves light, such as a pen light or flashlight. They were first asked if they play with their cat using an LLP, with response options including no, have never used; no, used to but no longer use; rarely (less than once a month); some (less than once a week); fair amount (2–3 times/week); frequently (more than three times a week); and daily. For those who did not currently use an LLP (not currently or ever), they were asked to select all reasons why they do not use them (e.g., “I am afraid of hurting my cat’s eyes”; “my cat does not like laser pointers”; etc.). Those guardians who indicated they currently do play with an LLP (or have in the past) were asked to select the reasons why (e.g., my cat seems to enjoy it; it is an easy way to play with my cat). They were then asked if they let their cat ‘catch’ something at the end of the play session (for example, by ending the game with the laser landing on a food treat or favorite stuffed mouse or toy). Additionally, they were asked how long the cat continues to look for the light when the game is over, and the cat’s typical behavior at the end of the LLP play session (e.g., goes to sleep; seems agitated or upset, etc.); and, if these behaviors are similar or different than when finished with other forms of play. They were asked if they feel their cat benefits from the LLP play, as well as if they think their cat suffers any negative effects of LLP play. All respondents were also asked to indicate, by selecting from a series of statements, what they had heard about LLPs (e.g., laser pointers are a good way to exercise your cat, laser pointers can lead to obsessive cat behavior).

In addition, respondents were asked to report the total amount of time they spend with their cat over the span of an entire day either playing with an LLP, in other forms of play, or cuddling/sitting together/petting, on a 7-point Likert scale (with 1 = less than 5 min and 7 = more than 5 h). Guardians were also asked to report how bonded they felt with their cat during each of these types of interactions using a 5-point Likert scale (with 1 = much less bonded, 5 = much more bonded).

Descriptive statistics were calculated to characterize guardians’ views of LLP play and their cats’ behaviors. Kruskal–Wallis (KW) nonparametric analyses of variance were used to explore the relationship between specific behaviors and LLP play. We next performed a multiple linear regression analysis using a total compulsive behavior score (the sum of reported compulsive behaviors significantly associated with LLP play) as the response variable. Results of the exploratory KW analysis were used to guide the selection of predictors for the multiple regression model. Potential predictor variables in the model included: LLP play [never used; used to use but no longer; rarely used (less than once a week); frequently used (at least 2–3 times a week)]; total number of cats living in the home (1, 2, >2); cat variables including age, sex, indoor/outdoor; guardian variables including age (18–29, 30–39, 40–49, 50 and older), gender, country, and bond score. Because LLP play was the largest predictor of potentially compulsive ARBs, separate chi-square analyses were next performed for each of the associated ARBs. Significance level (α) was set at *p* = 0.05 and all tests were two-tailed. Data were analyzed using SPSS (IBM, Armonk, NY, USA).

## 3. Results

### 3.1. Characteristics of Guardians and Cats (Descriptive Statistics)

A total of 618 responses were obtained, with the largest percentage from the United States (65.5%), followed by the United Kingdom (13.9%) and Canada (8.4%). The mean age of respondents was 39.8 (±12.3) years; median = 38 years and 88.5% identified as female. Most respondents had a university degree (78.6%).

Cat ages ranged from 1–2 years (15.9%) to older than 10 years of age (23.9%), and more than half of respondents (51.1%) had been living with their cat for five years or longer. The majority of cats in the study were either spayed females (48.6%) or neutered males (50.1%) and were classified as indoor cats (75.9%). Most cats were not declawed (93.5%). The median number of cats in respondents’ homes was 3, with 45.5% of respondents living in single-cat homes and 33.5% living in homes with two cats. The mean score for the affectionate companionship subscale of the Pet Relationship Scale (PRS) was 37.12 (± 7.0; range: 9–48). Demographic data on survey respondents and their cats are summarized in Table 1.

### 3.2. Cat Guardians’ Experience with Behavior Problems

A total of 553 (89.5%) of guardians reported their cat displayed at least one behavior problem and 5.8% reported that their cat had been treated by a veterinarian for an obsessive behavior. When asked if they had ever sought help for a specific behavior problem, 90 (14.6%) indicated they had sought help for “inappropriate” elimination/house soiling, 64 (10.4%) for excessive fear/anxiety, and 43 (7.0%) for aggression.

### 3.3. Reported Behaviors and Guardians’ Responses

**Figure 1:**
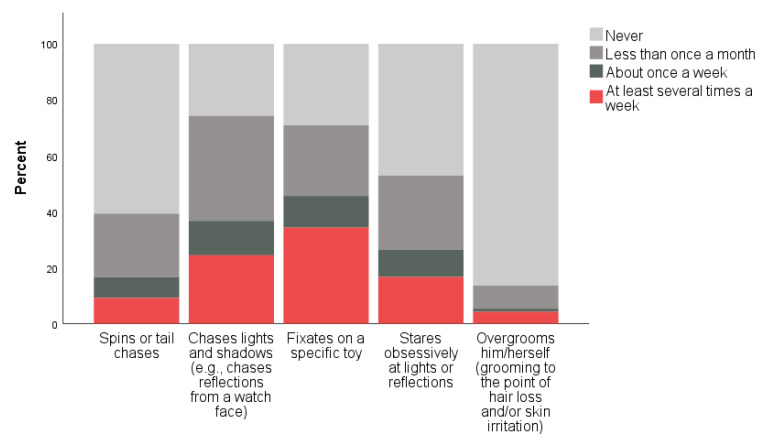
Cat guardian responses to questions related to frequency of exaggerated or abnormal repetitive behaviors in their cats.

**Figure 2:**
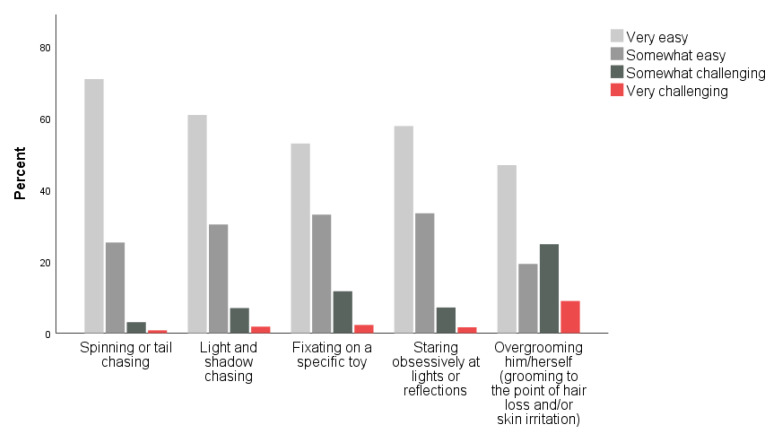
Cat guardian responses to questions related to their ability to redirect their cat’s behavior.

When asked to indicate how frequently their cat engaged in several behaviors that have been identified as potential indicators of FCD (including: spins or tail chases, chases lights and/or shadows, fixates on a specific toy, stares obsessively at lights or reflections, and overgrooming), the behaviors reported most frequently include fixating on a specific toy and chasing lights and/or shadows (Figure 1). When asked, for each behavior reported, how easy it was to redirect their cat when the cat is performing the behavior, the behaviors reported as easiest to redirect were spinning/tail chasing and light and/or shadow chasing (Figure 2).

**Figure 3:**
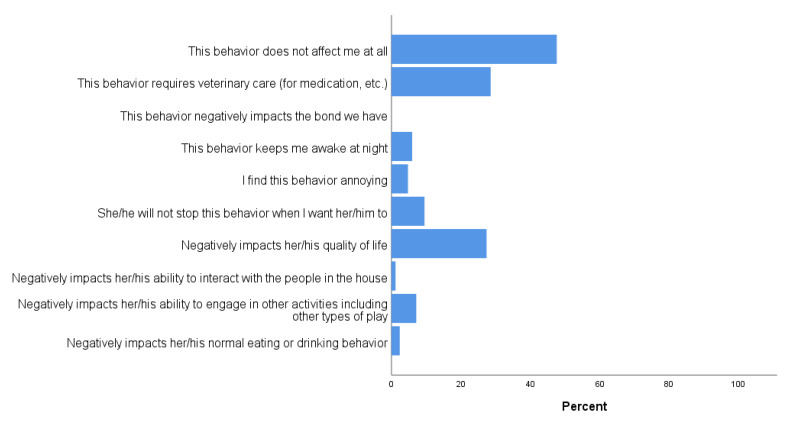
Cat guardian responses to questions related to the impact of overgrooming behaviors (*n* = 84).

In addition, respondents were asked to indicate the impact of each of these behaviors on their, or their cats’, lives. For those who reported spinning/tail chasing (*n* = 243), most guardians indicated that the behavior did not affect them at all (96.3%). A small percentage reported the behavior kept them awake at night (1.2%) or negatively impacted their cat’s quality of life (1.2%). The remaining effects (e.g., negatively impacts the guardian’s bond with their cat; requires medication; etc.) were reported by less than 1% of the respondents. Similarly, when asked about chasing lights or shadows (*n* = 459), 97.4% reported that the behavior did not affect them at all, and only 1.3% found the behavior annoying. The remaining effects were reported by less than 1% of the respondents. When asked about their cat fixating on a specific toy, 94.5% indicated the behavior did not affect them, similar to reports regarding staring at lights or reflections (*n* = 327, 95.4%). However, for overgrooming (*n* = 84), only 47.6% of guardians reported that the behavior did not affect them (Figure 3). Even so, no guardians reported that overgrooming negatively impacted the bond with their cat.

### 3.4. Cat Guardians’ Reported Use and Perceptions of Laser Light Pointers

**Figure 4:**
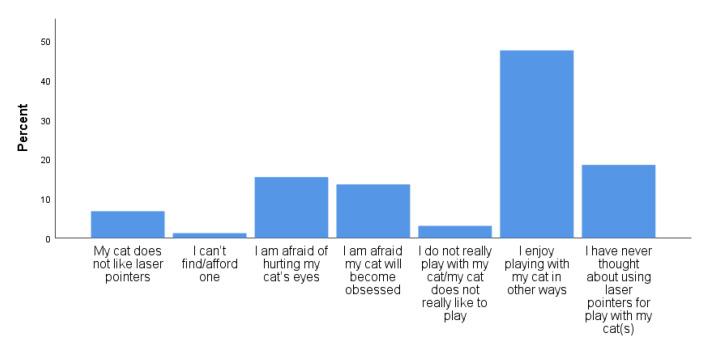
Reasons given by cat guardians for not currently (or ever) using laser light pointers to play with their cats (*n* = 336).

**Figure 5:**
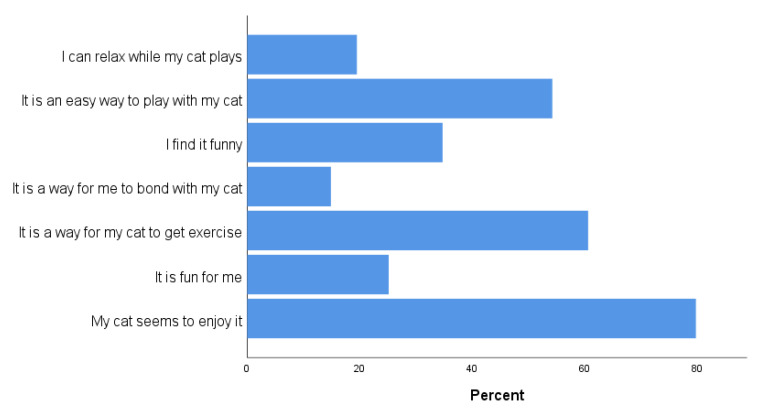
Reasons given by cat guardians for using laser light pointers to play with their cats (*n* = 282).

Over half of respondents did not currently use LLPs for cat play (54.5%), either because they have never used them (26.2%) or used them in the past but no longer (28.2%). Those who had never used, or no longer used an LLP were asked to indicate the reasons for their decision. The most common reason was that they enjoyed playing with their cat in other ways; only 5.2% reported that they were concerned about the development of obsessive behaviors (Figure 4). For those who currently used an LLP with their cat, 50.7% percent used them less than once a month, 29.1% less than once a week, 14.2% used them two to three times a week, 2.8% more than three times a week, and 3.2% reported using them daily. For regression and Kruskal–Wallis analysis, these response categories were combined into four categories: never used, used in past but no longer, used less than once a week, and used more than once a week. The guardians who reported using LLPs to play with their cat were asked to report the reasons why they used the LLPs. The most common response was that their cat seemed to enjoy it, followed by a way for their cat to get exercise (41.6%) and an easy way to play with their cat (39.0%) (Figure 5).

When guardians were asked to report the total amount of time they spent over the span of a day engaged in LLP play, other forms of play and cuddling/petting, they reported spending the least amount of time each day engaged in LLP play (Table 2). When asked how bonded they felt after interacting with their cat, guardians reported feeling most bonded after cuddling, followed by play without laser, and then LLP play (Table 3).

**Figure 6:**
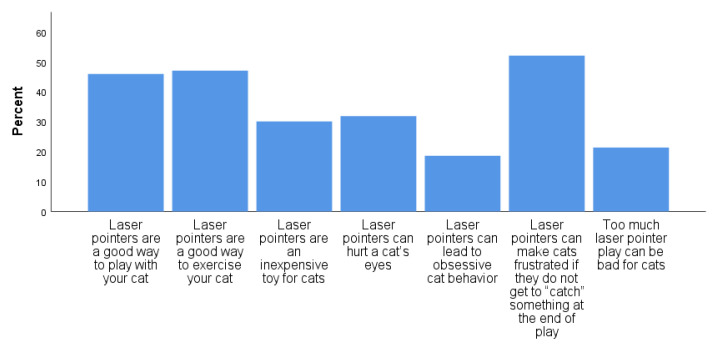
Laser light pointer statements reported to have been heard by cat guardians (*n* = 618).

When asked to indicate, from a series of statements about LLPs, those they had heard, the most commonly heard statements included ‘Laser pointers can make cats frustrated if they do not get to “catch” something at the end of play’ (reported to have been heard by 52.1%) and ‘Laser pointers are a good way to exercise your cat’ (47.1%) (Figure 6).

### 3.5. Associations between Exaggerated or Abnormal Repetitive Behaviors and Laser Light Play, Cat Guardian, Cat, and Household Variables

Kruskal–Wallis tests were performed to explore the relationship between LLP play (never used; used, but not currently; less than once a month; more than once a month) and frequency of ARBs (never; less than once a month; about once a week; at least several times a week). With the exception of overgrooming, all ARBs were significantly associated with LLP play (Table 4). The relationships between each feline ARB and frequency of LLP play are depicted in Table 5.

Multiple linear regression was conducted on the total ARB score to determine the impact of frequency of LLP play, while controlling for cat guardian factors (age, education, gender), cat factors (age, sex, indoor/outdoor, declaw status), and household variables (number of cats in the home). The multiple regression model predicting total ARBs using LLP play, guardian, cat and household factors was significant (F10 = 9.78, *p* < 0.001), with an *R*^2^ of 0.14. Significant predictors of feline ARB score included amount of LLP play (B = 0.79; *p* < 0.001), guardian age (B = −0.03; *p* = 0.027), cat age (B = −0.44; *p* < 0.001), and cat indoor/outdoor status (B = −0.65; *p* = 0.022). Complete results for the regression analysis are shown in Table 6, with the largest predictor of ARBs being LLP play.

Upon further analysis, guardians of indoor-only cats were more likely to report ARBs than indoor/outdoor cats (only one respondent reported their cat was an outdoor-only cat, so outdoor-only cats could not be assessed). One-way ANOVA was conducted to assess the relationship between guardian age and reported ARBs (F(3) = 7.16, *p* <0.001). Post hoc analysis (Fisher’s Least Significant Difference; LSD) found that guardians ages 18–29 were more likely to report ARBs than any other age group. No other differences between guardian ages were significant. One-way ANOVA was also used to determine the relationship between cat age and ARBs (F(4) = 7.89, *p* <0.001). Post hoc analysis found significant differences in reported ARBs for cats between 1 and 2 years of age compared to all older cats, with younger cats displaying more ARBs. Cats older than 10 years of age were the least likely to display ARBs, compared to younger cats.

Lastly, the results of the one-way ANOVA assessing LLP play and ARBs were significant (F(3.614) = 17.86, *p* <0.001). Post hoc analysis revealed a significant difference between each of the four categories (never used, used in past but no longer, used less than once a week, and used more than once a week). The guardians who reported never using an LLP to play with their cat were least likely to report ARBs, followed by those who used to, but no longer, use LLPs. Guardians who reported using an LLP less than once a month were more likely to report ARBs than those who did not use LLPs, but less likely than those who used an LLP more frequently. Those who reported using an LLP to play with their cat more than once a week were more likely to report ARBs than any other group of guardians.

## 4. Discussion

In this study, significant associations were found between the frequency of LLP play and the occurrence of all surveyed ARBs, with the exception of overgrooming, in owned companion cats. The strongest patterns were seen for behaviors which may be connected to LLP play: chasing lights or shadows, staring “obsessively” at lights or reflections, and fixating on a specific toy (Table 4 and Table 5). As these results are correlational, we cannot state that LLP play causes ARBs in companion cats; given the intrinsically rewarding nature of play, it may be that individual cats who enjoy laser play are motivated to look for more of this activity in other light sources or reflections (or vice versa). Over half of respondents who reported that their cat engaged in shadow or light chasing stated that it was easy to redirect the cat into another behavior, which could argue against a truly compulsive behavior in those cats (Figure 2). Nonetheless, frequency of LLP play was the strongest predictor of ARBs in the multiple regression model (Table 6), and guardians who frequently used LLPs for playing with their cats were more likely to report ARBs in their cats than others who did not use LLPs, or who used them less frequently (Table 5). While significant, the low R^2^ of the regression model likely reflects the high variability among both cats and their human guardians, and suggests that other intrinsic and extrinsic factors contribute to the development of ARBs in companion cats.

The lack of association between frequency of LLP play and overgrooming may be due to the diverse causes of this type of behavior in domestic cats. Grooming is a normal behavior in cats, and while grooming can also function as a displacement behavior during stress and conflict [22], underlying medical causes are often the primary cause of such self-directed trauma in cats [10,23]. It is perhaps also important that overgrooming was the behavior that guardians reported having the most difficulty in redirecting to other behaviors (Figure 2).

Indoor-only cats were more likely to display ARBs than cats allowed indoor/outdoor access (no outdoor-only cats were included in the data analysis). The link between outdoor access and reduced reports of ARBs cannot be explained with the data collected for this study, but possible contributing factors may include presence of stressors within the home for these cats (other cats, other animals, lack of enriched environment). Cats originally evolved to live an outdoor lifestyle, and indoor-only cats in particular may show increased stress from the presence of (incompatible) conspecifics, competition for resources, insufficient mental stimulation, and lack of physical exercise [24]. Provision of some form of outdoor access is recommended by some cat welfare organizations [25], and a number of studies have found that behavioral problems, such as unacceptable indoor elimination (often termed inappropriate elimination), destructive scratching, and aggression, as well as feline lower urinary tract disease (FLUTD) are more common in indoor-only cats vs. indoor/outdoor cats [20,26,27,28,29,30]. Compulsive behaviors are often seen in genetically-predisposed individuals exposed to chronic or recurrent stressors (motivational conflict, frustration, etc.), or whose behavioral needs are not adequately met [8,23].

Nearly half (45.5%) of the respondents in our study reported using LLPs to play with their cats; however, frequency of use was low for most survey respondents: approximately half (50.7%) of those who used LLPs with their cats, did so less than once a month. A small percentage of guardians, however, used LLPs frequently: 2.8% reported using them more than 3 times per week, and 3.2% reported using them daily. Similarly, when asked about how they interact with their cat regularly (e.g., by cuddling/petting, playing excluding LLP play, or playing with an LLP), guardians reported spending the least amount of time in LLP play: 73.8% reported spending less than 5 min per day in LLP play. In comparison, 62.1% of respondents reported playing in other ways with their cats for between 5 and 30 min per day, and 87.5% reported spending between 31 min and 5+ hours per day cuddling with or petting their cats.

Interestingly, the statement “Laser pointers can make cats frustrated if they do not get to ‘catch’ something at the end of play” was the statement reported as familiar to the greatest proportion of respondents (52.1%); yet only 35.6% of respondents reported following this advice. This was followed closely, however, by the statement “Laser pointers are a good way to exercise your cat” (47.1%), perhaps reflecting the current emphasis on providing both physical and mental exercise for companion cats, and partially explaining their use by a significant proportion of cat guardians. Only about one in five cat guardians reported familiarity with the statements “Laser pointers can lead to obsessive behavior” (18.6%) and “Too much laser pointer play can be bad for cats” (21.4%).

Many cats do, in fact, seem to enjoy LLP play; among guardians who did use LLPs to play with their cats, “My cat seems to enjoy it” was the most commonly reported reason why (54.0% of respondents). Guardians also commonly cited the exercise benefits for their cats, and the ease of playing with laser pointers with their cats, as reasons why they used these toys. The risk of development of compulsive behaviors was of less concern to cat guardians in the present study than was the risk of hurting their cat’s eyes (Figure 4), and only 5.2% of guardians reported the fear of their cat developing “obsessive” behaviors as their reason for not using laser play. The most common reason, by far, that guardians chose not to use laser pointers with their cats was “I enjoy playing with my cats in other ways.”

In the present study, 89.5% of cat guardians reported at least one potentially-problematic behavior in their cats. This percentage is in line with earlier work [31] in which cat guardians were asked to report whether certain behaviors, typically perceived as undesirable, were exhibited by their cat; note that these percentages are typically higher than those reported in studies looking at (for example) case reports from veterinary behavior practices. The behavior problems for which guardians most sought help included “inappropriate” elimination (14.6%), excessive fear/anxiety (10.4%), and aggression (7.0%). Only 5.8% reported that their cat had been treated by a veterinarian for an “obsessive” behavior, although this percentage is higher than reported in other studies (3.5% reporting compulsive behaviors in Amat et al. [20]; 3% reporting overgrooming/self-harming in Wassink-van der Schot et al. [32]). Nonetheless, cat guardians in this study appeared largely unbothered by these behaviors: ~94–97% reported the behaviors such as chasing lights or shadows, staring at lights or reflections, or fixating on a specific toy, did not bother them at all. The notable exception to this pattern was overgrooming: over half the respondents who reported this behavior stated that it did affect them, citing reasons such as “the behavior requires veterinary care (for medication, etc.)” and the behavior “negatively impacts (my cat’s) quality of life” (Figure 3). It is possible, given the link between serious behavior problems and relinquishment or euthanasia of pet cats, that cats with severe compulsive disorders were missing from our survey data; in which case, any existing relationship between LLP play and ARBs could be underestimated in our results. Despite this, however, less than 1% of respondents reported that any of these behaviors negatively impacted their bond with their cat, and no guardians reported that overgrooming impacted this bond. The mean score for the affectionate companionship subscale of the PRS for respondents in this study was 37.12 (range 9–48); this mean is higher than that reported for pet guardians in Kafer et al. [21] (30.24 for females and 27.88 for males).

Taken as a whole, our results suggest that LLP toys are used by a substantial proportion of cat guardians in the US, Canada and U.K. to play with their cats. However, frequency of use is relatively low, as is duration of time spent playing with LLP toys, when compared to time spent in other shared activities. Many guardians were familiar with potential risks of using LLP toys to play with their cats, and some (35.6%) followed the common advice to end the game by allowing their cat to “catch” a more tangible “prey” item. However, for many, the perceived (and possibly real) benefits to their cat (enrichment, physical exercise, enjoyment by the cat) appeared to outweigh any potential risks of this type of play.

It is concerning, therefore, that we did find a significant association between frequency of laser play and all forms of ARB, except overgrooming, and that LLP play was the largest predictor of ARBs. Overall, the more frequently LLP toys were used, the more likely guardians were to report ARBs. Although correlational, these results support concerns that LLP toys may be associated with the development of compulsive behaviors in cats, warranting further research into their risks.

Three possible limitations of the study should be considered. First, in common with many studies based on guardian reports, the ability of these guardians’ to correctly identify the behaviors asked about on the survey, and their accuracy in recollecting frequencies of play and behavior, may vary. For example, it is possible that some of the “compulsive” behaviors reported by guardians may have been learned/attention-seeking behaviors [9] seen only in the presence of the guardian; we do not have information on whether these cats also performed the behaviors outside of the guardians’ presence (i.e., supporting the true compulsive nature of the behaviors). Second, we did not investigate in detail other characteristics of guardians’ play styles with their cats (for example, other forms of active or hunting-type play, such as chasing string or feather wands), nor the timing of use of LLP play relative to when guardians’ first observed the ARBs; thus, we cannot comment on potential relationships between these types of play and development of ARBs in companion cats, including whether the reported ARBs predated or followed initial use of LLPs, as has been suggested elsewhere (14). We also cannot establish the ‘threshold’ at which laser play might be problematic. Third, the majority of respondents in the present study were women (88.5%) with a university degree (78.6%). This gender imbalance is a common challenge for survey-based studies which recruit participants through social media, as in the present study. Herzog [33] notes that women tend to show higher levels of positive behaviors and attitudes towards animals, although the effect sizes of gender differences in the human-animal attachment studies he reviewed ranged from none to small. Respondents in our study scored slightly higher on affectionate attachment than participants in an earlier study using the same scale; this may be due to the predominance of women in our study. If true, then it is possible that the other bond-related measures in our study (such as the fact that most guardians in our study reported that the ARBs did not bother them, and that these behaviors had essentially no negative impact on their bond with their cat) may be somewhat higher than in the general cat-owning population. This in turn would underscore concern for the impacts of laser light toys on frequency of some types of ARBs in pet cats.

## 5. Conclusions

Results of this study suggest an association between LLP play and the development of compulsive behaviors. Simply stated, the more frequently LLP toys were used, the more likely cat guardians were to report exaggerated or abnormal repetitive behaviors. These results support previously unsubstantiated concerns that LLP toys may be associated with the development of feline compulsive disorder. Further research into the risk of laser light pointer play is essential to better understanding this important component of companion cat care.

## Figures and Tables

**Table 1:** Demographic data on cat guardians and their cats (*n* = 618).

Human Participants					
Country	United States	United Kingdom	Canada	Australia	Other
	405 (65.5%)	86 (13.9%)	52 (8.4%)	10 (1.6%)	65 (10.5%)
**Age**	18–29 years	30–39 years	40–49 years	50 and older	
	149 (24.2%)	184 (29.9%)	137 (22.2%)	146 (23.7%)	
**Gender**	Female	Male	Non-binary	NA	
	547 (88.5%)	47 (7.6%)	21 (3.4%)	3 (0.5%)	
**Education**	High school/GED or College qualification (e.g., A/AS level, Nat cert/diploma)	Some college	University Degree (e.g., BS, BA, BSc/BSc (Hons))	Higher degree (e.g., MS, MA, MSc/PhD)	NA/Other
	44 (7.1%)	73 (11.8%)	196 (31.7%)	290 (46.9%)	15 (2.4%)
**Length of ownership**	6 months but less than 1 year	At least 1 year but less than 3 years	At least 3 years but less than 5 years	5 years or longer	
	32 (5.2%)	154 (24.9%)	116 (18.8%)	316 (51.1%)	
**Cats**					
**Cats in the home**	1	2	3	4	5 or more
	281 (45.5%)	207 (33.5%)	77 (12.5%)	27 (4.4%)	26 (4.3%)
**Age**	1–2 years	3–4 years	5–7 years	8–10 years	Older than 10 years
	98 (15.9%)	130 (21.0%)	157 (25.4%)	85 (13.8%)	148 (23.9%)
**Sex**	Male, neutered	Male, intact	Female, spayed	Female, intact	NA
	309 (50.1%)	4 (0.6%)	300 (48.6%)	4 (0.6%)	1
**Indoor/outdoor**	Indoor	Indoor/outdoor	Outdoor		
	469 (75.9%)	148 (23.9%)	1 (0.2%)		
**Declaw** **status**	Declawed, front only	Declawed, front and back	Not declawed		
	32 (5.2%)	8 (1.3%)	578 (93.5%)		

**Table 2:** Total amount of time spent over the span of an entire day in cat related activities.

	Play (Excluding Laser Light Play) (*n* = 564)	Laser Light Play (*n* = 290)	Cuddling/Petting (*n* = 586)
Less than 5 min	73 (12.9)	214 (73.8)	2 (0.3)
5–15 min	195 (34.6)	60 (20.7)	29 (4.9)
16–30 min	155 (27.5)	13 (4.5)	42 (7.2)
31–59 min	82 (14.5)	2 (0.7)	60 (10.2)
1–2 h	51 (9.0)	1 (0.3)	214 (36.5)
3–5 h	7 (1.2)	0	165 (28.2)
More than 5 h	1 (0.2)	0	74 (12.6)

**Table 3:** Guardian reported bond feelings after cat interactions.

	Play (Excluding Laser Light Play) (*n* = 574)	Laser Light Play (*n* = 297)	Cuddling/Petting (*n* = 582)
I feel much less bonded	1 (0.2)	7 (2.4)	3 (0.5)
I feel somewhat less bonded	5 (0.9)	15 (5.1)	0
No change in how bonded I feel	125 (21.8)	147 (49.5)	15 (2.6)
I feel somewhat more bonded	209 (36.4)	100 (33.7)	65 (11.2)
I feel much more bonded	234 (40.8)	28 (9.4)	499 (85.7)

**Table 4:** Kruskal–Wallis test results assessing the association between laser light play and exaggerated or abnormal repetitive behaviors (*n* = 561).

	H (df)	P
Spins or tail chases	7.89 (3)	=0.049
Chases lights and shadows	52.03 (3)	<0.001
Fixates on a specific toy	11.46 (3)	=0.009
Stares obsessively at lights or reflections	28.31 (3)	<0.001
Overgrooms him/herself	1.41 (3)	=0.704

**Table 5:** Incidence of exaggerated or abnormal repetitive behaviors and frequency of laser light play (*n* = 618).

	**Spins or Tail Chases**			
	**Never**	**Less Than Once a Month**	**About Once a Week**	**Several Times a Week**
Laser play				
Never used	114 (70%)	22 (14%)	12 (7%)	14 (9%)
Used to use	102 (59%)	51 (29%)	11 (6%)	10 (6%)
Less than once a month	125 (56%)	54 (24%)	20 (9%)	26 (12%)
More than once a month	34 (60%)	13 (23%)	3 (5%)	7 (12%)
	**Chases Lights and Shadows**			
	**Never**	**Less Than Once a Month**	**About Once a Week**	**Several Times a Week**
Never used	76 (47%)	56 (35%)	11 (7%)	19 (12%)
Used to use	38 (22%)	82 (47%)	18 (10%)	36 (21%)
Less than once a month	38 (17%)	81 (36%)	37 (16%)	69 (31%)
More than once a month	7 (12%)	13 (23%)	10 (18%)	27 (47%)
	**Fixates on a Specific Toy**			
	**Never**	**Less than ONCE a Month**	**About Once a Week**	**Several Times a Week**
Never used	63 (39%)	44 (27%)	13 (8%)	42 (26%)
Used to use	49 (28%)	44 (25%)	18 (10%)	63 (36%)
Less than once a month	55 (24%)	58 (26%)	30 (13%)	82 (36%)
More than once a month	13 (23%)	10 (18%)	9 (16%)	25 (44%)
	**Stares Obsessively at Lights or Reflections (*n* = 618)**			
	**Never**	**Less Than Once a Month**	**About Once a Week**	**Several Times a Week**
Never used	109 (67%)	27 (17%)	9 (6%)	17 (11%)
Used to use	78 (45%)	51 (29%)	22 (13%)	23 (13%)
Less than once a month	86 (38%)	73 (32%)	23 (10%)	43 (19%)
More than once a month	18 (32%)	13 (23%)	6 (11%)	20 (35%)
	**Overgrooming**			
	**Never**	**Less Than Once a Month**	**About Once a Week**	**Several Times a Week**
Never used	144 (89%)	12 (7%)	4 (3%)	2 (1%)
Used to use	148 (85%)	12 (7%)	2 (1%)	12 (7%)
Less than once a month	194 (86%)	21 (9%)	1 (0.5%)	9 (4%)
More than once a month	48 (84%)	6 (11%)	0	3 (5%)

**Table 6:** Results of the multiple linear regression model predicting feline abnormal repetitive behaviors (ARBs) as a function of cat, guardian and household factors.

ANOVA						
Model	Sum of Squares	df	Mean Squares	F	Sig.	
Regression Residual Total	848.53 5243.12 6091.66	10 604 614	78.92 8.68	9.78	<0.001	
Coefficients * (Dependent Variable: reported frequency of ARBs)						
Variable			Coefficient (B)	Std. Error	t	Sig.
(Constant)			11.08	1/26	8/80	<0.001
**Amount of laser (LLP) toy play**			**0.79**	**0.13**	**6.16**	**<0.001**
**Guardian age**			**−0.03**	**0.01**	**−2.51**	**0.027**
Guardian gender			0.34	0.25	1.34	0.180
Guardian education level			−0.19	0.12	−1.53	0.126
Cat sex			−0.08	0.12	−0.63	0.528
**Cat age**			**−0.44**	**0.12**	**−3.63**	**<0.001**
**Indoor/Outdoor**			**−0.65**	**0.29**	**−2.29**	**0.022**
Declaw status			−0.04	0.27	−1.6	0.880
Number of cats in household			−0.22	0.16	−1.36	0.176

* Significant predictors are shown in **Bold**.

## Data Availability

Data available by contacting the corresponding author.

## Supplementary Materials

The following are available online at https://www.mdpi.com/article/10.3390/ani11082178/s1, Survey.
