# Supplementary material for: Laser Light Pointers for Use in Companion Cat Play: Association with Guardian-Reported Abnormal Repetitive Behaviors
Source: Animals (Basel). 2021 Jul 23;11(8):2178. doi: 10.3390/ani11082178 (PMC8388446; doi:10.3390/ani11082178)
Supplement: Supplementary file 1 [file animals-11-02178-s001.zip › animals-1291250-supplementary.pdf]

Cats and Laser Pointer Play

Cat owners - Your experiences and perceptions are wanted. We are researchers who want to better understand how people interact and play with their cats. We are looking for cat owners at least 18 years of age who are the primary caretaker of an adult cat (at least one year old) and have owned this cat for at least six months. If you meet these qualifications, we would like to ask you to please consider taking the following short anonymous survey.

What is involved?
You will be asked to complete a series of questions as honestly as possible and there are no right or wrong answers. The questionnaire should take no more than 10 minutes to complete. Participation is entirely voluntary. You may quit at any time. This study has been approved by Colorado State University Research and Integrity Compliance and Review Board (#21-10566H).

Are there any benefits or risks in my taking part?
There are no direct risks or benefits to completing the survey. The survey is voluntary and anonymous and you may stop the survey at any time by closing the window. Data from the survey will be used only for research and will hopefully be published in a journal. Will my participation be confidential? Yes, all participation will be confidential. The data will be anonymous and will contain no information that could lead to the identity of individuals. Anonymous data will be kept on a password protected computer.

What happens if I change my mind?
If you feel you do not wish to continue with the questionnaire, you can close the browser window.

Where can I get more information?
If you have questions about this research please contact Dr. Lori Kogan (Lori.Kogan@ColoState.EDU). Any questions about participant rights related to this survey can be directed to CSU IRB (ricro_irb@mail.colostate.edu) or 970 491-1655. Questions about the survey can be directed to Lori Kogan, PhD at lori.kogan@colostate.edu.

I have read and understood the information given above. In consenting, I agree to take part in this research project and agree for my data to be used for the purpose of this study. I understand that my participation is voluntary and I may withdraw at any time.

- ◯ Yes, I agree to take part in this study
- ◯ No, I do not agree to take part in this study

Please tell us a little about you.

In what country do you currently live?

- ◯ United States
- ◯ United Kingdom
- ◯ Canada
- ◯ Australia
- ◯ Other (please specify): ________________________________

*

What is your age?

____

How do you identify yourself?

- ◯ Female
- ◯ Male
- ◯ Non-binary/other
- ◯ Prefer not to say

Please select the highest level of education you have completed:

- ◯ High school/GED or College qualification (e.g. A/AS level, Nat cert/diploma)
- ◯ Some college
- ◯ University Degree (e.g. , BS, BA, BSc/BSc (Hons))
- ◯ Higher degree (e.g., MS, MA, MSc/PhD)
- ◯ Prefer not to say
- ◯ Other

How many cats do you have in your home?

▼ 0 - No cats in the home ... More than 10

Are you the primary caretaker for at least one cat in your home?

- ◯ Yes
- ◯ No

NOTE -- If you have more than one cat, please answer the following survey questions for the cat whose name begins with the letter that comes first in the alphabet. For example, if you have a cat named Fluffy and another cat named Roy, please answer the survey about Fluffy, since “F” comes before “R” in the alphabet.

How old is your cat?

- ○ Less than 12 months
- ○ 1-2 years old
- ○ 3-4 years old
- ○ 5-7 years old
- ○ 8-10 years old
- ○ Older than 10 years of age

What is the sex of your cat?

- ○ Male, not neutered
- ○ Male, neutered
- ○ Female, not spayed
- ○ Female, spayed
- ○ Don't know

How long have you owned your cat?

- Less than 6 months
- 6 months but less than 1 year
- At least 1 year but less than 3 years
- At least 3 years but less than 5 years
- 5 years or longer

Would you classify your cat as:

- Indoor
- Indoor/outdoor
- Outdoor

Please indicate which best describes your cat:

- Declawed, front only
- Declawed, front and back
- Not declawed

Please indicate your agreement level to the following statements:

	Strongly disagree	Moderately disagree	Mildly disagree	Mildly agree	Moderately agree	Strongly agree
There are times I'd be lonely except for my cat	○	○	○	○	○	○
My cat and I watch/stream TV (e.g., Netflix, Hulu, etc.) together frequently	○	○	○	○	○	○
I give gifts to my cat for birthdays and special occasions	○	○	○	○	○	○
My cat is valuable to me	○	○	○	○	○	○
I talk to my cat about things that bother me	○	○	○	○	○	○
Making me laugh is part of my cat's job	○	○	○	○	○	○
I miss my cat when I am away	○	○	○	○	○	○
My cat gives me reason for getting up in the morning	○	○	○	○	○	○
I consider my cat a member of the family	○	○	○	○	○	○
I have considered relinquishing	○	○	○	○	○	○

my cat because of his/her behavior						
My cat provides me comfort during difficult times	○	○	○	○	○	○

Please indicate how often your cat exhibits the following behaviors:

	Never	Less than once a month	About once a month	About once a week	Several times a week	Daily	Multiple times a day
Spins or tail chases	○	○	○	○	○	○	○
Chases lights and shadows (e.g., chases reflections from a watch face)	○	○	○	○	○	○	○
Fixates on a specific toy	○	○	○	○	○	○	○
Stares obsessively at lights or reflections	○	○	○	○	○	○	○
Overgrooms him/herself (grooming to the point of hair loss and/or skin irritation)	○	○	○	○	○	○	○

How does the behavior **overgrooming him/herself (grooming to the point of hair loss and/or skin irritation)** impact you and your cat? Please select all that apply.

- ☐ Negatively impacts her/his normal eating or drinking behavior
- ☐ Negatively impacts her/his ability to engage in other activities including other types of play
- ☐ Negatively impacts her/his ability to interact with the people in the house
- ☐ Negatively impacts her/his quality of life
- ☐ This behavior does not affect me at all
- ☐ She/he will not stop this behavior when I want her/him to
- ☐ I find this behavior annoying
- ☐ This behavior keeps me awake at night
- ☐ This behavior negatively impacts the bond we have
- ☐ This behavior requires veterinary care (for medication, etc.)

How does the behavior **spinning or tail chasing** impact you and your cat? Please select all that apply.

- ☐ Negatively impacts her/his normal eating or drinking behavior
- ☐ Negatively impacts her/his ability to engage in other activities including other types of play
- ☐ Negatively impacts her/his ability to interact with the people in the house
- ☐ Negatively impacts her/his quality of life
- ☐ This behavior does not affect me at all
- ☐ I find this behavior annoying
- ☐ She/he will not stop this behavior when I want her/him to
- ☐ This behavior keeps me awake at night
- ☐ This behavior negatively impacts the bond we have
- ☐ This behavior requires veterinary care (for medication, etc.)

How does the behavior **light and shadow chasing** impact you and your cat? Please select all that apply.

- ☐ Negatively impacts her/his normal eating or drinking behavior
- ☐ Negatively impacts her/his ability to engage in other activities including other types of play
- ☐ Negatively impacts her/his ability to interact with the people in the house
- ☐ Negatively impacts her/his quality of life
- ☐ This behavior does not affect me at all
- ☐ I find this behavior annoying
- ☐ She/he will not stop this behavior when I want her/him to
- ☐ This behavior keeps me awake at night
- ☐ This behavior negatively impacts the bond we have
- ☐ This behavior requires veterinary care (for medication, etc.)

How does the behavior **fixation on a specific toy** impact you and your cat? Please select all that apply.

- ☐ Negatively impacts her/his normal eating or drinking behavior
- ☐ Negatively impacts her/his ability to engage in other activities including other types of play
- ☐ Negatively impacts her/his ability to interact with the people in the house
- ☐ Negatively impacts her/his quality of life
- ☐ This behavior does not affect me at all
- ☐ I find this behavior annoying
- ☐ She/he will not stop this behavior when I want her/him to
- ☐ This behavior keeps me awake at night
- ☐ This behavior negatively impacts the bond we have
- ☐ This behavior requires veterinary care (for medication, etc.)

How does the behavior **staring obsessively at lights or reflections** impact you and your cat? Please select all that apply.

- ☐ Negatively impacts her/his normal eating or drinking behavior
- ☐ Negatively impacts her/his ability to engage in other activities including other types of play
- ☐ Negatively impacts her/his ability to interact with the people in the house
- ☐ Negatively impacts her/his quality of life
- ☐ This behavior does not affect me at all
- ☐ I find this behavior annoying
- ☐ She/he will not stop this behavior when I want her/him to
- ☐ This behavior keeps me awake at night
- ☐ This behavior negatively impacts the bond we have
- ☐ This behavior requires veterinary care (for medication, etc.)

Has your cat ever been treated by the veterinarian (e.g., prescribed medication, made to wear an Elizabethan collar (plastic cone around the neck) or surgical recovery suit) for any kind of obsessive behavior (e.g., spinning/chasing tail, staring obsessively, etc.)?

- ○ No
- ○ Yes (Please describe): ____

This is a test question, for this question, please select option C.

- A
- B
- C
- Not any of the above

Have you ever sought help for any of the following behavior problems with this cat?

	Yes	No
Excessive fear/anxiety		
Aggression		
Inappropriate elimination/house soiling		

How easy is it to redirect your cat when he/she is doing the following behaviors?

	Very easy	Somewhat easy	Somewhat challenging	Very challenging	Not applicable
Spinning or tail chasing	○	○	○	○	○
Light and shadow chasing	○	○	○	○	○
Fixating on a specific toy	○	○	○	○	○
Staring obsessively at lights or reflections	○	○	○	○	○
Overgrooming him/herself (grooming to the point of hair loss and/or skin irritation)	○	○	○	○	○

For the following questions, please note the term ‘laser pointer’ and ‘laser pointer play’ includes any form of play that involves light - such as a pen light or flashlight.

Do you play with any of your cats using a laser pointer?

- ◯ No, I've never used
- ◯ Yes, I used to use, but I no longer do
- ◯ Yes, I currently use rarely (less than once a month)
- ◯ Yes, I currently use some (less than once a week)
- ◯ Yes, I currently use a fair amount (2-3 times a week)
- ◯ Yes, I currently use frequently (more than 3 times a week)
- ◯ Yes, I currently use daily

Why do you not play with your cats using a laser pointer? (Select all that apply).

- ☐ My cat does not like laser pointers
- ☐ I can’t find/afford one
- ☐ I am afraid of hurting my cat’s eyes
- ☐ I am afraid my cat will become obsessed
- ☐ I do not really play with my cat/my cat does not really like to play
- ☐ I enjoy playing with my cat in other ways
- ☐ I have never thought about using laser pointers for play with my cat(s)
- ☐ Other (please explain) ____

Why do you not currently play with your cats using a laser pointer? (Select all that apply).

- ☐ My cat does not like laser pointers
- ☐ I can't find/afford one
- ☐ I am afraid of hurting my cat's eyes
- ☐ I am afraid my cat will become obsessed
- ☐ I think my cat was becoming or had become obsessed
- ☐ I do not really play with my cat/my cat does not really like to play
- ☐ I enjoy playing with my cat in other ways
- ☐ I have never thought about using laser pointers for play with my cat(s)
- ☐ It seemed to lead to negative behaviors (please explain): ____
- ☐ Other (please explain) ____

Please indicate which of the following statements you have heard about playing with your cat using laser pointers (select all that apply):

- ☐ Laser pointers are a good way to play with your cat
- ☐ Laser pointers are a good way to exercise your cat
- ☐ Laser pointers are an inexpensive toy for cats
- ☐ Laser pointers can hurt a cat's eyes
- ☐ Laser pointers can lead to obsessive cat behavior
- ☐ Laser pointers can make cats frustrated if they do not get to "catch" something at the end of play
- ☐ Too much laser pointer play can be bad for cats
- ☐ Other: ____

Please indicate the total amount of time you spend for each of the following activities with your cat over the span of an entire day:

	Less than 5 minutes	5-15 minutes	16-30 minutes	31-59 minutes	1-2 hours	3-5 hours	More than 5 hours	Not applicable	Don't know
Any type of play EXCEPT laser pointer play	○	○	○	○	○	○	○	○	○
Playing with your cat using a laser pointer	○	○	○	○	○	○	○	○	○
Cuddling/sitting together/petting	○	○	○	○	○	○	○	○	○

Please indicate the length of a typical play session with your cat:

	Less than 5 minutes	5-9 minutes	10-19 minutes	20-29 minutes	1-2 hours	3-5 hours	More than 5 hours	Not applicable	Don't know
Any type of play EXCEPT laser pointer play	○	○	○	○	○	○	○	○	○
Playing with your cat using a laser pointer	○	○	○	○	○	○	○	○	○

Please indicate how bonded you feel with your cat during the following types of interactions:

	I feel much less bonded	I feel somewhat less bonded	No change in how bonded I feel	I feel somewhat more bonded	I feel much more bonded	Not applicable
Any type of play EXCEPT laser pointer play	○	○	○	○	○	○
Playing with your cat using a laser pointer	○	○	○	○	○	○
Cuddling/sitting together/petting	○	○	○	○	○	○

This is a test question, for this question, please select option B.

- ○ A
- ○ B
- ○ C
- ○ Not any of the above

Why do you (or did you) play with your cat using a laser pointer? (select all that apply):

- ☐ My cat seems/seemed to enjoy it
- ☐ It is/was fun for me
- ☐ It is/was a way for my cat to get exercise
- ☐ It is/was a way for me to bond with my cat
- ☐ I find/found it funny
- ☐ It is/was an easy way to play with my cat
- ☐ I can/could relax while my cat plays
- ☐ Other: ____

Do you (or did you) usually end your laser pointer play session by letting your cat ‘catch’ something (e.g., land on a stuffed toy, give them a treat, etc)?

- ◯ No
- ◯ Yes, please explain: ____
- ◯ I don't know/I don't remember

How long does (or did) your cat continue looking for the laser light when game is over?

- ◯ No time at all
- ◯ 1-2 minutes
- ◯ 3-5 minutes
- ◯ 6-10 minutes
- ◯ Over 10 minutes
- ◯ Varies – please explain ____
- ◯ I don't know/I don't remember

Please describe your cat’s typical behavior after a play session using a laser pointer:

- ◯ Goes to sleep
- ◯ Walks away and does something else
- ◯ Stares at the laser pointer or where she last saw it
- ◯ Seems agitated or upset (e.g.., tail flicks, dilate pupils, jumpy/nervous)
- ◯ Seems relaxed
- ◯ Behaves aggressively towards people or other pets in the home
- ◯ Other (please explain): ____
- ◯ I don't know/I don't remember

Are the behaviors you described after a laser pointer play session similar or different than when he/she finishes other types of play (such as with a stuffed toy, wand toy, etc.)?

- ◯ Similar
- ◯ Different (please explain how):
____
- ◯ I don't know/I don't remember

Do you (or did you) think your cat benefits from playing with a laser pointer?

- ◯ No
- ◯ Yes (please explain) ____

Do you think your cat has suffered any negative effects from playing with a laser pointer?

- ○ No
- ○ Yes (please explain) ____

Additional comments about play with your cat:

____

Thank you for your time.
